# Delayed Double Treatment with Adult-Sourced Adipose-Derived Mesenchymal Stem Cells Increases Striatal Medium-Spiny Neuronal Number, Decreases Striatal Microglial Number, and Has No Subventricular Proliferative Effect, after Acute Neonatal Hypoxia-Ischemia in Male Rats

**DOI:** 10.3390/ijms22157862

**Published:** 2021-07-23

**Authors:** Haylee K. Basham, Benjamin E. Aghoghovwia, Panagiotis Papaioannou, Steve Seo, Dorothy E. Oorschot

**Affiliations:** Department of Anatomy, School of Biomedical Sciences, and the Brain Health Research Centre, University of Otago, P.O. Box 913, Dunedin 9054, New Zealand; basha736@student.otago.ac.nz (H.K.B.); aghbe756@student.otago.ac.nz (B.E.A.); pappa302@student.otago.ac.nz (P.P.); steve.seo@otago.ac.nz (S.S.)

**Keywords:** adipose-derived mesenchymal stem cells, adult mesenchymal stem cells, location of extrinsic stem cells in brain, striatal medium-spiny projection neurons, striatal microglia, subventricular zone, cell proliferation, stereology

## Abstract

Perinatal hypoxia-ischemia (HI) is a major cause of striatal injury. Delayed post-treatment with adult-sourced bone marrow-derived mesenchymal stem cells (BMSCs) increased the absolute number of striatal medium-spiny neurons (MSNs) following perinatal HI-induced brain injury. Yet extraction of BMSCs is more invasive and difficult compared to extraction of adipose-derived mesenchymal stem cells (AD-MSCs), which are easily sourced from subcutaneous tissue. Adult-sourced AD-MSCs are also superior to BMSCs in the treatment of adult ischemic stroke. Therefore, we investigated whether delayed post-treatment with adult-sourced AD-MSCs increased the absolute number of striatal MSNs following perinatal HI-induced brain injury. This included investigation of the location of injected AD-MSCs within the brain, which were widespread in the dorsolateral subventricular zone (dlSVZ) at 1 day after their injection. Cells extracted from adult rat tissue were verified to be stem cells by their adherence to tissue culture plastic and their expression of specific ‘cluster of differentiation’ (CD) markers. They were verified to be AD-MSCs by their ability to differentiate into adipocytes and osteocytes in vitro. Postnatal day (PN) 7/8, male Sprague-Dawley rats were exposed to either HI right-sided brain injury or no HI injury. The HI rats were either untreated (HI + Diluent), single stem cell-treated (HI + MSCs×1), or double stem cell-treated (HI + MSCs×2). Control rats that were matched-for-weight and litter had no HI injury and were treated with diluent (Uninjured + Diluent). Treatment with AD-MSCs or diluent occurred either 7 days, or 7 and 9 days, after HI. There was a significant increase in the absolute number of striatal dopamine and cyclic AMP-regulated phosphoprotein (DARPP-32)-positive MSNs in the double stem cell-treated (HI + MSCs×2) group and the normal control group compared to the HI + Diluent group at PN21. We therefore investigated two potential mechanisms for this effect of double-treatment with AD-MSCs. Specifically, did AD-MSCs: (i) increase the proliferation of cells within the dlSVZ, and (ii) decrease the microglial response in the dlSVZ and striatum? It was found that a primary repair mechanism triggered by double treatment with AD-MSCs involved significantly decreased striatal inflammation. The results may lead to the development of clinically effective and less invasive stem cell therapies for neonatal HI brain injury.

## 1. Introduction

Perinatal hypoxia-ischemia (HI) can lead to acute brain damage and is a major risk factor for the development of cerebral palsy [1]. The medium-spiny projection neurons (MSNs) of the dorsal striatum (i.e., caudate putamen, CPu), which constitute >97% of the resident neurons [2], are among the most injured cerebral neurons following acute HI in both the rat and human [3,4,5,6]. Injury to the CPu is manifested as movement impairments that are characteristic of cerebral palsy [3,4,6].

The Rice–Vannucci rat HI model [3] is a well-established animal model to induce and mimic cerebral palsy. Using the classic, or modified, version of this model, studies have focused on the prevention and cure of the brain injury. Existing therapeutic strategies aim to either protect, or replace and restore, injured brain tissue. Neuroprotective therapies include moderate hypothermia, antioxidants, and glutamate antagonists [5,7,8,9,10]. They are, however, only effective if administered within 0–6 h after HI-induced brain injury, thus providing a short or narrow window for treatment [8]. In contrast, neurorestorative interventions have a delayed onset, generally involve treatment with stem cells, and are effective after neonatal HI brain injury even though the onset of treatment is delayed for days. For example, the administration of a high dose of bone marrow-derived mesenchymal stem cells (BMSCs, i.e., 750,000–1,000,000 BMSCs per rat), one week after neonatal HI brain injury, increased the absolute number of MSNs in the CPu compared to treatment with diluent [6]. Other studies have shown that delayed treatment with BMSCs enhances proliferation and/or survival of newly formed cells of host origin in the cerebral cortex and hippocampus following neonatal HI brain injury [11,12,13].

During the past decade, mesenchymal stem cells (MSCs) have been extensively investigated for their therapeutic potential after brain injury [14]. For perinatal hypoxic-ischemic brain injury relevant to term infants, BMSCs have been extensively investigated ([6,11,12,13], see also Table 1 in [15]). However, extraction of BMSCs is more invasive and difficult compared to extraction of adipose-derived MSCs (AD-MSCs), which are easily sourced from subcutaneous tissue and thus clinically more advantageous as a source of stem cells [16]. Adult-sourced AD-MSCs are also superior to BMSCs in the treatment of adult ischemic stroke in mice [17], possibly due to a <10% difference in their secretion profile of growth factors [17,18]. AD-MSCs also showed higher proliferative activity after 1–2 days of culturing, with greater production of vascular endothelial cell growth factor and hepatocyte growth factor than BMSCs [17]. There are no reported preclinical studies, however, on the effects of AD-MSCs on perinatal hypoxic-ischemic brain injury [15] and, to our knowledge, no studies since 2017. Furthermore, the optimal source of MSCs for the treatment of perinatal hypoxic-ischemic brain injury remains to be determined [15].

Therefore, the first major aim of the study was to investigate whether delayed post-intervention with a high dose (i.e., 1,070,000 cells, on average), and repeated high doses, of adult-sourced AD-MSCs increased the absolute number of striatal MSNs in the rat, compared to treatment with diluent, following neonatal HI. To investigate this aim, it was first necessary to confirm that the cultured AD-MSCs exhibited characteristics of stem cells, namely adherence to plastic in vitro [19], expression of stem cell cluster of differentiation (CD) markers, and differentiation into adipocytes and osteocytes in vitro [20,21]. It was also necessary to confirm that the injected MSCs were present in the brain and to investigate where they were specifically located [22].

In the literature on the treatment of perinatal HI at term-equivalent, and of adult stroke, various mechanisms have been indicated by which MSCs could be effective. These can be summarized as (i) MSCs enhance neurogenesis (of the endogenous neural stem cell population in the subventricular zone (SVZ), for example, [23]). It should be noted that ‘enhance neurogenesis’ is better described as ‘enhance cellular proliferation’ in most studies since specific *neuronal* markers were not used to identify the cells of interest in the SVZ [11,12,13,24,25,26,27,28,29]; (ii) MSCs enhance the subsequent differentiation of these progenitor cells into neurons, oligodendroglia and astrocytes in the damaged brain tissue; (iii) MSCs modulate or dampen the local immune response involving microglia and T lymphocytes/cells [14,15,30]. We investigated, therefore, the effect of AD-MSCs in perinatal HI on key aspects of these three potential mechanisms as our second major aim. Specifically, we investigated (i) cellular proliferation in the SVZ, (ii) the differentiation of progenitors into MSNs in the CPu, and (iii) the response of microglia in the SVZ and the CPu.

## 2. Materials and Methods

This study was approved by the Committee on Ethics in the Care and Use of Laboratory Animals at the University of Otago.

### 2.1. Culturing of MSCs

Male Sprague-Dawley rats (200–350 g) were euthanized with CO_2_. Using sterile procedures, their deep lateral inguinal, inguinal, and the abdominal fat pads were harvested, washed and minced in sterile 25 mL Dulbecco’s phosphate buffered saline (DPBS) plus 1% antibiotic-antimycotic solution. The minced tissue was then digested with 0.075% collagenase under gentle agitation every 5 min for 60–75 min at 37 °C [31] and centrifuged at 400× *g* for 5 min. The resulting cell pellet was homogenized in growth medium (i.e., 10% fetal bovine serum, FBS) and 1% antibiotic-antimycotic solution in Dulbecco’s Modified Eagle’s Medium (DMEM, low glucose), filtered through a sterile 100 µm filter into a sterile petri-dish, and transferred into a new sterile centrifuge tube. Cells were then plated in 25 cm^2^ vented culture flasks (i.e., onto tissue culture plastic) and incubated at 37 °C for 24 h in a sterile incubator containing 5% CO_2_.

For the first experiment, a pilot study was conducted to determine whether a batch of FBS effectively supported proliferation of MSCs derived from adipose tissue. A specific batch of FBS supported cell proliferation into 95–100% confluency at an average of 2.67 ± 0.82 days, for 3 passages. This proliferation rate was similar to previously cultured BMSCs that, when injected in vivo, significantly increased the absolute number of striatal MSNs after neonatal HI [6]. Hence, the same batch of FBS from the pilot study was used to culture the MSCs derived from adipose tissue for all the experiments reported herein.

After 24 h for all experiments, the seeding medium was replaced with freshly prepared growth medium, and again after 72 h if the cells were not 90–100% confluent. When confluent, the cells were trypsinized/passaged. Passaging was carried out for 3–5 times to ensure a purified population of MSCs [6,32] prior to injection into the rat pups. For each passage, the cells were plated onto tissue culture plastic. The cells adhered well to the tissue culture plastic, fulfilling one of the characteristics of stem cells [19].

**Figure 1:**
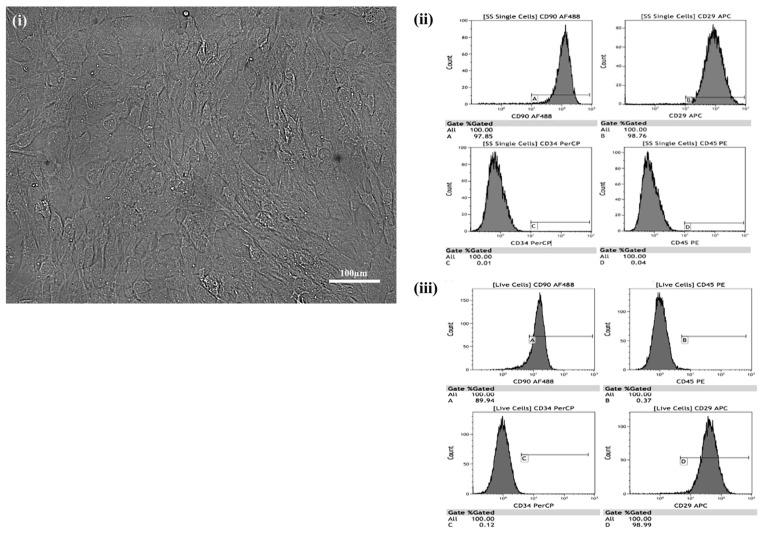
Characterization of cultured adipose-derived-mesenchymal stem cells (AD-MSCs). (**i**) Phase-contrast image. (**ii**, **iii**) Fluorescence-activated cell analysis (**ii**, **A**–**D**), including live cell analysis (**iii**, **A**–**D**), of cluster of differentiation (CD) expression (dark grey) by cultured (passage 4) adult-sourced AD-MSCs.

Prior to injecting cultured cells into the animals, they were analyzed using a rat multipotent mesenchymal stem cell marker antibody panel [33] and a fluorescence-activated cell (FACS) analyzer [6,12] to confirm that the majority of injected cells were MSCs and were alive. Briefly, five antibodies were used (CD90 (Bio Legend, San Diego, CA, USA, Cat#202505), CD29 (Bio Legend, Cat#102215), CD45 (Bio Legend, Cat#202207), CD34 (Santa Cruz Biotechnology, Santa Cruz, CA, USA, Lot#F0413) and IgG (Bio Legend, Cat#407405)). The antibodies were incubated individually, or altogether for the CD antibodies, with the cultured MSCs. The cells were then investigated for the expression of surface markers using a FACS analyzer as previously described [6]. The vast majority of cells (Figure 1(ii)) expressed the stem cell markers CD90 (97.85% positive) and CD29 (98.76% positive). They expressed the non-stem cell markers, CD34 and CD45, in very small percentages (0.01% and 0.04%, respectively, Figure 1(ii)).

To verify that the process of trypsinization prior to in vivo injection yielded live MSCs, cells were prepared as described above for stem cell characterization using CD markers. They were then incubated in a solution of yellow fluorescent reactive dye (live/dead stain, diluted 1:1000 µL PBS) (Lot#1780387, Eugene, OR, USA) for 30 min as a final step. Gating-based flow cytometric analysis confirmed that a high percentage of the stem cells were live stem cells positive for CD90 (89.94% positive) and CD29 (98.99% positive, Figure 1(iii)).

### 2.2. Differentiation of Cultured MSCs

A subset of confluent cultured cells at passage number six was investigated for their differentiation potential. Poly-D-lysine (5 mg in 50 mL sterile Milli-Q water, [P6707], Sigma-Aldrich, St Louis, MO, USA) was used to promote adhesion of potentially differentiating cells to the base of culture flasks. One mL of this solution was added per 25 cm^2^ flask (yielding 4 µg/cm^2^ of poly-D-lysine). After 15 min, the solution was removed by aspiration and the surface thoroughly rinsed with Milli-Q water (3 × 3 mL). The flasks were then dried for 2.5 h at 37 °C in an incubator. Then, 7 mL of standard medium and 1 mL of trypsinized cells at passage six was added to three poly-D-lysine-coated 25 cm^2^ flasks. The trypsinized cells were from a confluent 75 cm^2^ flask, with the cells cultured in standard medium. This procedure was repeated three times to yield nine seeded 25 cm^2^ flasks, three per experimental condition. One additional confluent 25 cm^2^ flask at passage number five, that was not subsequently grown on poly-D-lysine, was also used in this experiment as a control culture. The presence or absence of poly-D-lysine did not affect the experimental outcome (see below). To investigate the differentiation of adipocytes and osteocytes in the 25 cm^2^ flasks, 4 mL of the standard medium was removed after 24 h and immediately replaced with 4 mL of differentiation-specific medium (i.e., PromoCell mesenchymal stem cell adipogenic differentiation medium 2 (C-28016) for adipocytes and PromoCell mesenchymal stem cell osteogenic differentiation medium (C-28013) for osteocytes). For the control cultures, 4 mL of new standard growth medium was added. Every third day the procedure was repeated, with 4 mL of the current medium removed and 4 mL of the respective differentiation or control medium added. Cell cultures in adipocyte differentiation medium were cultured for 16/17 days (in triplicate flasks), while those in osteocyte differentiation medium were cultured for 21 days (in triplicate flasks) [34]. Concurrent control cultures were analyzed at either 16/17 days or 21 days in culture (in duplicate flasks for each time point).

### 2.3. Staining to Detect Cultured Stem Cell Differentiation

**Figure 2:**
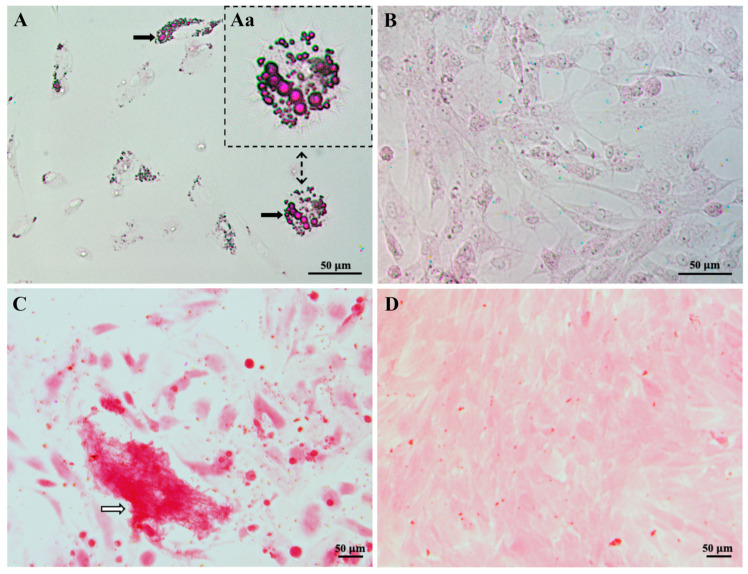
Differentiation of cultured mesenchymal stem cells (MSCs). All images are brightfield. In (**A**) MSCs that had differentiated into adipocytes after incubation in specific adipocyte differentiation media for 16/17 days are shown. Black arrows indicate red lipid droplets stained by an Oil Red O solution. (**Aa**) is a magnified area of (**A**) displaying differentiated adipocytes. In (**B**) MSCs that were maintained in normal growth media as a control for (**A**) showed no stained lipid droplets. In (**C**) MSCs that had differentiated into osteocytes after incubation in specific osteocyte differentiation media for 21 days are shown. A calcium deposit is indicated by a white arrow. In (**D**) MSCs at culture passage 12 are shown. These cells were maintained in the growth media as a control for (**C**). Note that the small red dots are floating precipitates of the stain and not stained cells. Since the cultured MSCs were derived from adipose tissue, and could differentiate into adipocytes or osteocytes, they specifically were adipose-derived (AD)-MSCs.

At either culture day 16/17 or 21, the cell culture medium was removed, and the cells were gently washed (2 × 3 mL DPBS) at 37 °C. Eight mL of formalin (10%, pH 7) was added to the cells by pipetting onto the wall of the flask and gently rotating the flask while ensuring not to add formalin directly to the cells. Cells were then incubated for 30 min at 37 °C in an incubator. Thereafter formalin was discarded, and the cells were washed (Milli-Q ×2). To detect adipogenic differentiation, the cells grown in adipocyte differentiation or control medium were incubated in 60% isopropanol for 5 min. The 60% isopropanol was then discarded, and the cells were covered evenly with Oil Red O Working solution for 10–20 min. The cells were then washed (Milli-Q ×3) or until no excess stain was visible. To detect osteocyte differentiation, the cells grown in osteocyte differentiation medium or control medium, were fixed in formalin and then washed in Milli-Q water as described above. They were then incubated in Alizarin Red stain for 20 min. Thereafter the cells were washed (Milli-Q ×5) or until no excess stain was observed. For both the adipocyte and osteogenic experiments, the cells were then covered in Milli-Q water to avoid the cells drying out and photographed on an IX71 Olympus phase-contrast microscope. Lipid droplets were red in differentiated adipocytes (Figure 2A) and nuclei were purple for undifferentiated adipocytes in control cultures (Figure 2B). Calcium deposition was red/orange in cells exposed to osteocyte differentiation medium (Figure 2C) compared to control cell cultures (Figure 2D) [34]. Since the cultured cells were able to differentiate into different cell types (i.e., adipocytes, Figure 2A; osteocytes, Figure 2C), these results confirmed that the cultured cells were stem cells. As the cultured stem cells were derived from adipose tissue, they specifically were AD-MSCs.

### 2.4. Neonatal HI and Treatment with AD-MSCs

**Figure 3:**
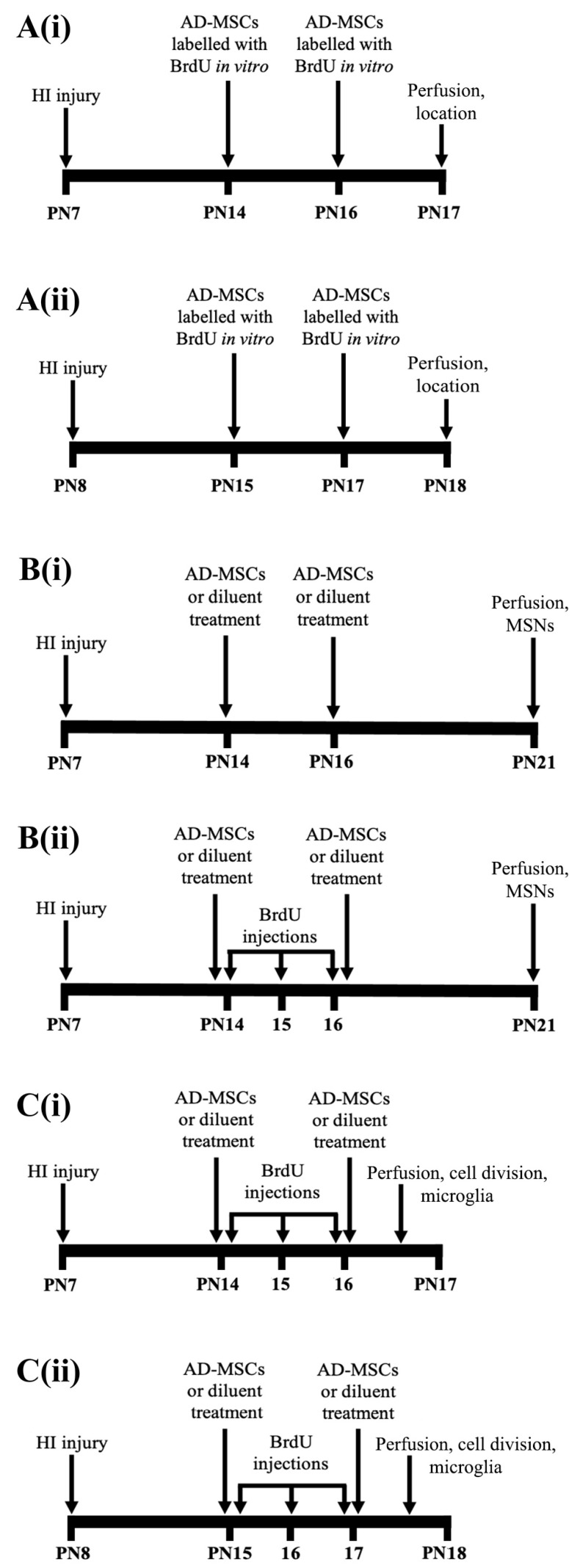
Timeline for each experiment after neonatal hypoxia-ischemia (HI) of the brain. (**A**) Location of injected adipose-derived-mesenchymal stem cells (AD-MSCs) in the brain (**A**(i), postnatal day (PN) 7–17; **A**(ii), PN8–18). (**B**) Effect of treatment with AD-MSCs on the absolute number of all medium-spiny neurons (MSNs, **B**(i)), and the absolute number of progenitors that had differentiated into MSNs (**B**(ii)), in the caudate-putamen (CPu) at PN21. (**C**) Effect of treatment with AD-MSCs on cellular proliferation in the dorsolateral subventricular zone (dlSVZ) and the absolute number of microglia in the CPu and dlSVZ (**C**(i), PN7–17; **C**(ii), PN8–18). BrdU, bromodeoxyuridine.

A total of eight Sprague-Dawley rat litters were obtained from the University of Otago Hercus Taieri Resource Unit. Each litter contained a dam and 10 pups, with female pups being preferentially culled in larger litters. The pups’ day of birth was designated as postnatal day (PN) 0 and exposure to neonatal HI occurred on PN7 or PN8 (Figure 3A–C). One litter of pups was exposed on PN8 due to a logistical constraint (i.e., PN7 for this litter was a Sunday). Immediately prior to the start of surgery on PN7 or PN8 for neonatal HI (see below), the male pups in each litter were weighed and matched-for-weight in either pairs, triplets or quadruplets in accordance with each specific experiment (see below). This specific matching was maintained throughout each experiment. Forty matched-for-weight male rats were used in total across three different experiments.

To increase comparability with other studies in the field, a modified version of the Rice–Vannucci model of acute HI injury was used on PN7 or PN8 [3,5,6,9,32,35]. Each HI pup was anesthetized with 5% halothane (for induction, 0.8% for maintenance) in oxygen (O_2_) and nitrous oxide (N_2_O). The right common carotid artery was double-ligated with a sterile 5–0 silk suture, followed by nail clipping for identification. Control (non-HI) pups were anesthetized and nail-clipped also. Thirty min after the last surgery, all pups from a litter were returned to the dam to suckle for 2 h [5,6,9]. HI pups were then exposed to hypoxia of 8% O_2_/92% nitrogen for 90 min in a 37 °C waterbath [3,5,6,9,35]. After hypoxia, pups recovered for 20 min before being returned to their dam. All pups were weighed daily from PN7/8 to PN17/18 for the location, cellular proliferation, and microglial experiments (see below), and until PN21 for the differentiation/medium-spiny neuron (MSN) experiment (see below), to monitor their health and well-being.

For all experiments outlined below, rats were injected subcutaneously at the back of the neck [6] with either a high dose [6,11,13] of AD-MSCs (956,000–1,245,000 (average 1,070,000) cells in 0.3 mL per injection) or with diluent. Stem-cell-treated rats received either single or double treatment with AD-MSCs (see Figure 3 and the text below for specific details). The diluent consisted of sterile Dulbecco’s phosphate buffered saline (DPBS) containing 0.5% bovine serum albumin (BSA) (i.e., DPBS/BSA). All stem-cell-injected rats were injected with AD-MSCs in sterile DPBS/BSA.

The first in vivo experiment investigated the location of injected AD-MSCs in the brain. Five male rats from two separate litters (2–3 pups per litter) received double stem cell-treatment (AD-MSCs×2) of subcutaneously injected bromodeoxyuridine *(BrdU)-labeled AD-MSCs*, on PN14 and PN16, or PN15 and PN17, followed by perfusion on PN17 or PN18, respectively (Figure 3A(i,ii)). The cultured AD-MSCs were confluent (70–95%) passage 5 cells that had been incubated in normal tissue culture medium containing 10 µM BrdU for 1–3 days prior to their subcutaneous injection into a rat. The BrdU was included in the tissue culture medium from the time of the previous trypsinization of these cultured AD-MSCs. Incubation in BrdU allowed its incorporation into the dividing cultured AD-MSCs, with the injected BrdU-labeled AD-MSCs subsequently detected immunohistochemically (see below) in the brain of each rat pup.

The second in vivo experiment investigated the effect of single and double treatment of AD-MSCs on the absolute number of MSNs in the CPu at PN21 (Figure 3B(i,ii)). PN21 was chosen to allow time for any newly formed cells to differentiate into MSNs. Twenty-seven male rats from five separate litters (4–8 pups per litter) were used. One of these litters contained littermates that were in the second litter of the previous experiment on the location of injected AD-MSCs. For the PN21 experiments, male pups were weight-matched as a set of quadruplets in four of the litters. For the fifth litter, male pups were weight-matched as either a set of quadruplets or a set of triplets, with *n* = 7 male pups in the litter. In each quadruplet, rats were subjected to either HI right-sided brain injury or no HI injury, and assigned to one of the following groups: (1) HI and untreated (HI + Diluent), (2) HI and single stem cell-treatment (HI + AD-MSCs×1), (3) HI and double stem cell-treatment (HI + AD-MSCs×2) or, (4) no HI injury and treatment with diluent (i.e., this was a normal control pup (Uninjured + Diluent)). For the triplet, the uninjured diluent-treated condition was omitted. The pups were injected on PN14 and PN16 (Figure 3B(i,ii)), with Group 1 and Group 4 rats injected with diluent on both days. Group 2 rats were injected with AD-MSCs on PN14 and diluent on PN16, and Group 3 rats were injected with AD-MSCs on both days. Eleven pups from two litters (4–7 pups per litter) were also injected with BrdU (25 mg/kg) to track the differentiation of newly generated progenitors (Figure 3B(i)). Specifically, BrdU was injected to track the migration and proliferation of daughter neuroblasts from the dlSVZ into the injured CPu [6,36,37,38,39]. Pups were injected with BrdU on PN14, PN15 and PN16 (Figure 3B(ii)). Sixteen pups from three of the litters (4–8 pups per litter) were not injected with BrdU (Figure 3B(i)) but were stereologically analyzed for the absolute number of MSNs.

The third in vivo experiment investigated the effect of AD-MSCs on cellular proliferation in the SVZ (specifically, the dorsolateral SVZ, dlSVZ), and the absolute number of microglial cells in the dlSVZ and the CPu. Eight different male rats, compared to the previous experiments, from three separate litters (2–4 pups per litter) were used. One of these litters contained littermates that were in the first litter of the experiment on the in vivo location of injected BrdU-labeled cultured AD-MSCs. Within each litter, pairs of male pups were weight-matched on either PN7 or PN8 and then randomly assigned to a treatment group. These rats received either double stem cell-treatment (AD-MSCs×2) or double diluent treatment, on PN14 and PN16 (Figure 3C(i)), or on PN15 and PN17 (Figure 3C(ii)). These rats also received daily intraperitoneal injections with BrdU (25 mg/kg, Sigma-Aldrich, St. Louis, MO, USA) on PN14,15,16 (Figure 3C(i)), or PN15,16,17 (Figure 3C(ii)), to label dividing cells intrinsically located in the dlSVZ. This timing was chosen because neonatal rat hypoxia-ischemia-induced neurogenesis starts in the dlSVZ at 2 days after injury and continues for at least 2 months [36,37,40,41]. In addition, a microglia response to neonatal rat hypoxia-ischemia commences in the brain within 24 h and persists for up to 42 days [42,43]. Hence, the effect of the AD-MSCs, given at 7 and at 9 days post-injury, on cellular proliferation and microglia could realistically be investigated. For the microglial experiment, BrdU was not required to investigate the effect of AD-MSCs on microglial cells following neonatal HI. Instead, the same rat pups used for the microglial experiment were also used to investigate cellular proliferation in the dlSVZ and this second component required BrdU injections. The injection with BrdU occurred approximately 30 min after the injection with either AD-MSCs or diluent. The injection volume was 0.1 mL per 10 g of body weight. The rats were then perfused on PN17 and PN18, respectively (Figure 3C(i,ii)), to investigate the short-term response within the brain of treatment with AD-MSCs.

### 2.5. Immunohistochemistry

At either PN17/18 or PN21 (Figure 3), each rat was anesthetized with ketamine/xylazine (200 and 18 mg/kg, respectively), and intracardially perfused with 4% paraformaldehyde in 0.1 M phosphate buffer (pH 7.2). Each brain was partitioned with a Gem blade into the right and left cerebrum and hindbrain, weighed, and then cryoprotected in 30% sucrose, frozen in dry ice and stored at −80 °C.

Serial coronal 5 µm-thick sections of every right cerebral hemisphere were systematically sampled, after a random start, during sectioning on a cryostat. Two pairs of every 30th section were collected (e.g., sections 25/26, 27/28; 55/56, 57/58) onto 3-aminopropyltriethoxysilane-coated, or Trajan Series 2 adhesive (Trajan Scientific and Medical, Ringwood, Victoria, Australia), slides. Each slide contained one pair of sections. The second pair usually served as back-up sections, as did the second section of the first pair. For the microglial experiment, the second pair of sections were used. For the MSN experiment, two pairs of every 40th section were collected for three of the five litters (because initial analyses of the number of sections sampled per brain indicated that this sampling interval was sufficient for these litters). Pairs of sections were collected from before the start of the anterior pole of the CPu to beyond when the anterior commissure was no longer present in the midline [6]. Thus, the entire anterior CPu, and the dlSVZ and associated neural stem cell niche (NSCN) within the same sections, was sampled. This particular span was chosen because the bulk of migrating SVZ progenitor cells are obtained from the anterior SVZ and reside along the adjacent CPu [44,45]. In one sampled subregion containing the CPu, a third pair of sections was also collected to serve as a negative control. Each primary antibody used had its own negative control section pair per rat. For immunohistochemistry, one of every two pairs of sections was selected, along with the negative control sections. All subsequent processing was completed at room temperature unless otherwise indicated.

Sections affixed to slides were washed in 0.1 M phosphate buffer solution ((PBS) 3 × 5 min) and then incubated in 50% ethanol (30 min) to increase antibody permeability [46]. Sections were then washed (PBS, 3 × 5 min) and incubated in 4 M hydrochloric acid (HCl, 10 min; for the location, progenitor MSN, and cellular proliferation experiments only) at 37 °C. HCl denatured/unfolded the nuclear DNA. Denaturing facilitated increased exposure of the BrdU that had been incorporated into newly synthesized DNA of any dividing cells and thus enhanced subsequent binding of the anti-BrdU primary antibody. After washes (PBS, 3 × 5 min), all sections were incubated in 5% heat-inactivated goat serum (Hercus Taieri Resource Unit) in PBS (60 min) to block non-specific binding sites. Sections were then incubated overnight at 4 °C in either monoclonal mouse anti-BrdU primary antibody (for the location, progenitor MSN, and cellular proliferation experiments; RPN202, Amersham, 1:50) in antibody diluting buffer ((ADB) 1% BSA in 0.1 M PBS), with 0.1% Tween, or in rabbit polyclonal anti-ionized calcium-binding adaptor protein 1 (IBA-1) primary antibody (019-19741, WAKO, 1:1000) in ADB. The IBA-1 primary antibody was used to detect microglia. The negative control sections were incubated in only ADB with Tween (for the BrdU experiments) or in only ADB (for the IBA-1 experiment). Tween was added to the primary antibody and negative control solution to reduce non-specific binding and to heighten the infiltration of the reagents by functioning as a surfactant.

The following day all sections were washed (PBS, 3 × 5 min) and incubated in either a biotinylated anti-mouse secondary antibody (for the location, progenitor MSN, and cellular proliferation experiments; BA-9200, Vector Laboratories, 1:200) or a biotinylated anti-rabbit secondary antibody (for the microglial experiment; BA-1000, Vector Laboratories, 1:200) in ADB with 5% goat serum (60 min). After washes (PBS, 3 × 5 min), all sections were incubated in 0.3% hydrogen peroxide (H_2_O_2_) in PBS (10 min) to remove endogenous peroxidase activity and then washed (PBS, 3 × 5 min) again. All sections were then incubated in a tertiary antibody, streptavidin-conjugated horseradish peroxidase (SA-5004, Vector Laboratories, 1:200, 60 min).

**Figure 4:**
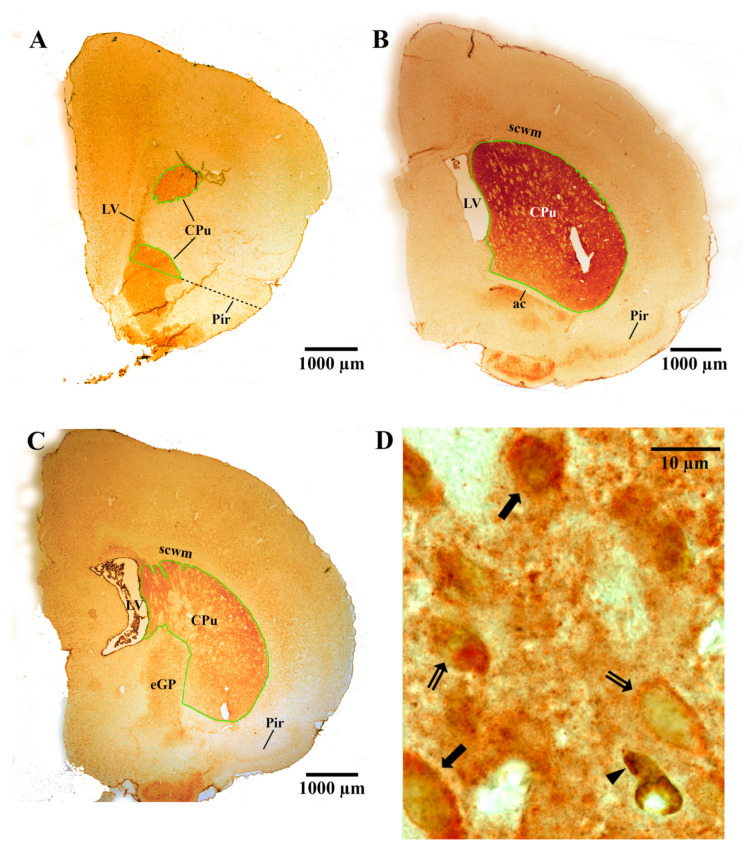
Representative images of the caudate-putamen (CPu), including immunostained medium-spiny neurons. (**A**–**C**) Light microscopic images of the CPu from a postnatal day (PN) 21 rat pup that was exposed to hypoxia-ischemia (HI) at PN7 and treated with adipose-derived-mesenchymal stem cells (AD-MSCs; **A**,**B**) or diluent (**C**) on PN14 and PN16. The CPu is specifically delineated with a green contour to measure the reference volume, Vref. The dotted black line in (**A**) is used to determine the inferior boundary (see also the text in the Methods). (**D**) Light microscopic image of medium-spiny neurons (i.e., dopamine and cyclic AMP-regulated phosphoprotein (DARPP-32)-positive neurons) in the CPu of a HI diluent-treated PN21 rat. The DARPP-32-positive neurons appeared as orange-colored cells (i.e., they had DARPP-32-positive orange staining in their cytoplasm from immunostaining with 3-amino-9-ethylcarbazole). Some of the neurons had large visible unstained nuclei (indicated by the double-tailed arrows). DARPP-32-positive/bromodeoyxyuridine (BrdU)-positive medium-spiny neurons had brown-stained nuclei due to BrdU immunostaining with diaminobenzidine (indicated by the solid black arrows). Note that the cell indicated at the black arrowhead is labeled with BrdU only. ac, anterior commissure; eGP, external globus pallidus; LV, lateral ventricle; Pir, piriform cortex; scwm, subcortical white matter.

**Figure 5:**
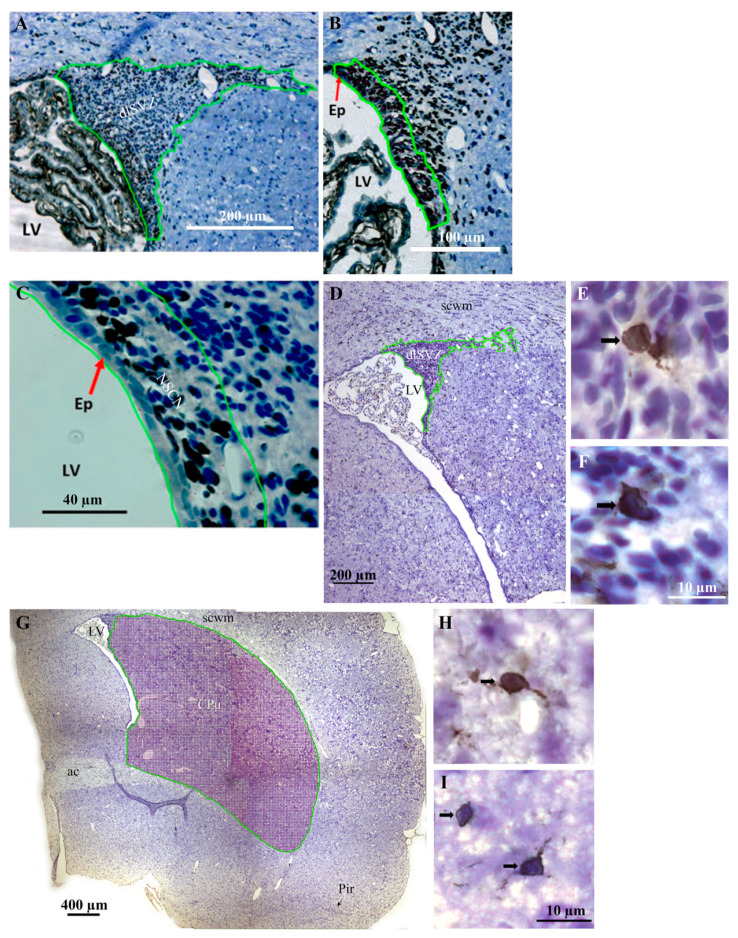
Representative brightfield images of the dorsolateral subventricular zone (dlSVZ), the neural stem cell niche (NSCN) region of the dlSVZ, and the caudate-putamen (CPu), including immunostained cells (e.g., microglia). (**A**) shows the inferomedial boundary of the dlSVZ, and (**B**,**C**) illustrate the NSCN region of the dlSVZ, in a postnatal day (PN) 17 rat pup exposed to hypoxia-ischemia (HI) on PN7. (**B**) is shown at a higher magnification in (**C**). Note in (**C**) that the NSCN region is ~4 cell diameters adjacent to the ependymal (Ep) cell lining of the lateral ventricle (LV). Bromodeoxyurdine-positive cells are stained black due to immunostaining using diaminobenzidine. Other cells and tissues are stained blue due to counterstaining with thionin. (**D**,**G**) Outline of the dlSVZ (**D**) and CPu (**G**) in a posterior coronal section from the brain immunostained with the microglial marker, anti-ionized calcium-binding adaptor protein 1 (IBA-1), and counterstained with thionin (blue). The section is from a PN17 rat pup that was exposed to HI at PN7 and treated with cultured adipose-derived-mesenchymal stem cells (AD-MSCs) on PN14 and PN16. (**E**,**F**) An image of an IBA-1 positive microglial cell in the dlSVZ of a HI AD-MSCs×2 (double)-treated (**E**) and HI diluent-treated (**F**) rat pup. (**H**,**I**) An image of an IBA-1 positive microglial cell in the CPu of a HI AD-MSCs×2-treated (**H**) and HI diluent-treated (**I**) rat pup. Black arrows indicate microglial cells, which had a pale nucleus that was surrounded by an IBA-1-positive dark brown/black circumferential, or almost circumferential, soma as a result of immunostaining using diaminobenzidine. ac, anterior commissure; cc, corpus callosum; Pir, piriform cortex; scwm, subcortical white matter.

After the sections were washed (PBS, 2 × 3 min) the antigen/antibody complex in the sections was visualized with 20 mg 3,3′-diaminobenzidine tetrahydrochloride (DAB; D5905, Sigma-Aldrich) in 40 mL PBS, 200 µL nickel chloride and 2.4 µL of 15% H_2_O_2_ (for the location experiment and for the brain sections from six rats for the cellular proliferation experiment) or by dissolving 37 mg of DAB powder (=20 mg of DAB substrate, D5637, Sigma-Aldrich) in 40 mL of PBS, 200 μL nickel chloride and 24 μL of 15% H_2_O_2_ (for the progenitor MSN and microglial experiments). The DAB yielded a black/brown reaction product in immunolabeled cells (Figure 4D and Figure 5C,E,F,H,I). The sections (except for those used for the progenitor MSN experiment, which underwent further processing for immunohistochemistry, see below) were then counterstained with 0.05% thionin to allow better visualization of the subependymal and ependymal layer of the lateral ventricle (LV), blood vessels and the boundaries of the CPu. Sections were then washed in distilled water for 20 s, followed by incubation in ascending concentrations of ethanol (70%, 95%, 100%, 100%, 20 s each) to dehydrate the sections. Then xylene was applied by soaking all sections in two successive 100% xylene baths (4 min each). All sections were coverslipped with dibutylphthalate polystyrene xylene (DPX) mounting media. The antigen/antibody complexes in the brain sections from two rats at the start of the cellular proliferation experiment were visualized by incubation in 3-amino-9-ethylcarbazole (AEC; Sigma, USA) in acetate buffer (70 mL) and 15% H_2_O_2_ (150 μL). This yielded a red reaction product in immunolabeled cells. These sections were then washed with tap water (2 × 5 min) and once with PBS (1 × 5 min) before being coverslipped in glycerol gelatin (Sigma-Aldrich, USA). All sections were then analyzed stereologically (see below).

For the MSN experiment, sections from 11 rats underwent double-immunolabeling for BrdU and a specific MSN cytoplasmic protein called dopamine and cyclic AMP-regulated phosphoprotein (i.e., DARPP-32 [47]). After incubation in DAB, sections to be double-immunostained were washed (PBS, 3 × 5 min) and incubated in 5% goat serum (60 min), followed by an overnight incubation at 4 °C in rabbit anti-DARPP-32 (1:200, #2302, Cell Signaling Technology, Danvers, MA, USA) in ADB with 0.1% Tween. Negative control sections were incubated in only ADB with Tween. Sections from another 16 rats underwent single-immunolabeling for DARPP-32. These sections were sequentially incubated in 50% ethanol, PBS, goat serum, and then the primary DARPP-32 antibody, as described above. The next day, the sections were washed (PBS, 3 × 5 min) and incubated in biotinylated anti-rabbit IgG (Vector Laboratories, Burlingame, CA, USA, 1:200 in DB without Tween, 60 min). Thereafter the sections were rinsed (PBS, 3 × 5 min), incubated in 0.3% H_2_O_2_ (10 min), washed (PBS, 4 × 5 min) and then incubated in streptavidin-conjugated horseradish peroxidase (1:200 in ADB without Tween, Vector Laboratories, 60 min). After the sections were washed (PBS, 3 × 2 min), they were incubated in AEC in acetate buffer for 30 min to visualize the antigen/antibody complex. The AEC yielded a red reaction product in the neuronal cytoplasm of MSNs, specifically within the striatum (Figure 4). The sections were then washed with tap water (2 × 5 min) and once with PBS (1 × 5 min) before being coverslipped in glycerol gelatin for subsequent stereological analyses.

After treatment with BMSCs, the findings for MSNs in the CPu were the same from two different methods (i.e., immunohistochemical staining for DARPP versus histochemical cresyl violet staining [6]. Hence, measurement of the absolute number of striatal DARPP-positive immunostained neurons was the focus of the current study.

### 2.6. Stereological Analyses

All sections were coded prior to stereological analyses. The absolute number (*N*) of various cell types was measured: (i) DARPP-32-positive and BrdU-DARPP-32-positive striatal MSNs within the CPu, (ii) BrdU-positive proliferating cells within the NSCN of the dlSVZ, and (iii) IBA-1-positive microglial cells in the entire dlSVZ and CPu. *N* was obtained by multiplying the total (i.e., absolute) reference volume (Vref) of the region of interest by the average number of cells in a subvolume (Nv) [6,48,49,50,51]. Thus, *N* = Vref × Nv.

#### 2.6.1. Measurement of the Vref

To measure the Vref of the CPu, dlSVZ or NSCN, Cavalieri’s estimator was used [6,48]. First, the boundaries of the CPu, dlSVZ or NSCN were defined in each sampled serial section of every rat. The boundary of the CPu was defined using Plates 12–37 of ref. [52] and the descriptions of ref. [49]. Specifically, the superior, lateral and medial boundaries of the CPu were defined using the LV and the white matter surrounding the structure (Figure 4A–C). For the inferior border of the CPu, in the most anterior sections, this was demarcated by a line from the inferior pole of the LV to the superior part of the medial bulge of the piriform cortex (Figure 4A; see also Figure 2A in ref. [49]). More posteriorly, the textural difference between the accumbens nucleus core and the CPu was also used to define the inferior boundary of the CPu at the anterior parts of the CPu (Figure 4A). When the anterior commissure was located inferior to the LV, or close to the LV as it travels from lateral to medial as one proceeds posteriorly, then the texture of the tissue located inferiorly and medially, between the LV and the anterior commissure, guided whether it was the CPu or not. When the anterior commissure was close to the midline, its lateral extent was used as the inferior boundary of the CPu (Figure 4B and Figure 5G). Even more posteriorly, the textural difference between the external globus pallidus (eGP) and the CPu was also used to define the boundary of the CPu at its inferior and medial parts (Figure 4C).

**Figure 6:**
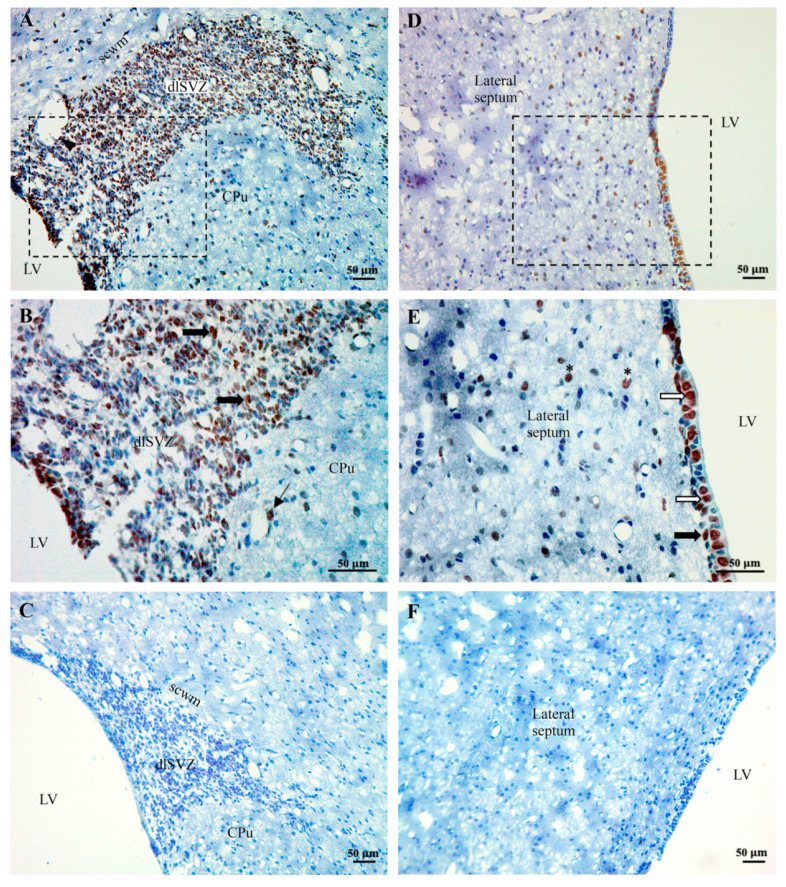
Brightfield images showing the location of subcutaneously injected cultured adipose-derived-mesenchymal stem cells (AD-MSCs) in the rat brain. (**A**,**B**) In the dorsolateral subventricular zone (dlSVZ) and caudate-putamen (CPu), bromodeoyuridine (BrdU)-labeled AD-MSCs were immunostained with diaminobenzidine yielding black/brown cells. They are indicated with either a single thick black arrow (dlSVZ) or a thin black arrow (CPu). The boxed area in (**A**) is shown at higher magnification in (**B**). The black arrowhead in (**A**) indicates a blood vessel. (**D**,**E**) BrdU-labeled AD-MSCs were also evident in the ependymal layer and subventricular zone (SVZ) of the lateral ventricle (LV) and the lateral septum. The boxed area in (**D**) is shown at higher magnification in (**E**). In (**E**), BrdU-labeled AD-MSCs are indicated by a white single arrow in the ependymal layer, and by a black arrow in the subependymal layer, of the SVZ, and by an * in the lateral septum. Note that there were numerous labeled AD-MSCs in the dlSVZ and the ependymal layer and fewer labeled AD-MSCs in the CPu and lateral septum. (**C**,**F**) Negative control sections showing no BrdU-labeled AD-MSCs. All sections are from a postnatal day (PN) 17 rat that was subcutaneously injected with cultured BrdU-labeled AD-MSCs on PN14 and PN16, following perinatal hypoxia-ischemia (HI) at PN7. All sections were counterstained with thionin (blue). scwm, subcortical white matter.

The dlSVZ was defined as the densely stained tissue that spanned laterally from the superolateral wall of the lateral ventricle (LV, Figure 5A,D and Figure 6A,B,E). The lateral wall of the lateral ventricle, including its ependymal layer (Figure 5B,C), was the medial boundary of the dlSVZ. The dlSVZ then extended laterally, first forming triangular-shaped tissue and then a tapering tail (Figure 5A,D and Figure 6A,C). Throughout its extent it was located between the subcortical white matter superiorly and medially and the CPu inferiorly and laterally (Figure 5D and Figure 6A,C). In sections where the expanded dlSVZ extended superiorly, and then medially to the LV, its superomedial border was defined by drawing a horizontal line from the most superior point of the LV cavity to the medial edge of the dlSVZ. The inferolateral boundary of the dlSVZ was defined by drawing a horizontal line at the location where the dlSVZ commenced its obvious expansion laterally (Figure 5A,B). The greater width of this lateral expansion was compared to the inferiorly located, constantly thinner, width of the SVZ (see Figure 6E) that was positioned immediately lateral to the lateral ventricle. It is acknowledged that Figure 6E is of the inferomedial wall of the LV, yet the inferolateral wall of the LV exhibited a very similar structure (i.e., appearance). In the anterior-most section of each brain, where the LV could be small, and the relatively small dlSVZ was difficult to distinguish from the rest of the small SVZ, the entire SVZ was outlined as the dlSVZ.

The boundary of the NSCN was defined in serial sections based on the most medial SVZ (i.e., the SVZm) in Figure 1 of ref. [36]. Hence, the NSCN was located medially in the dlSVZ, adjacent to the LV (Figure 5B,C). It extended vertically along the inferior-superior extent of the dlSVZ (Figure 5B). It extended laterally as a narrow ‘4 nuclear cell diameter’ margin ([36]; Figure 5C). In the current study, this margin was 41.8 µm in width (i.e., 18.6 mm width on a computer monitor at a magnification of ×445 using a ×20 objective).

The Vref of each specific structure for each rat was estimated using a computerized Cavalieri’s estimator (e.g., Figure 5G) as previously described [6,50]. After a random start, a systematic sampling interval was used (e.g., every 30th–60th pair of coronal sections) through the regions of interest at PN17/18 and PN21 (see Table 1 for the specific details for each brain region of interest). All sections were sampled from the start of the anterior pole of the CPu to the last section that contained both the anterior commissure in its medial midline location and the more laterally positioned CPu [6,52]. Thus, an exhaustive sampling of the entire anterior striatum was performed, but only the dorsal striatum (i.e., superior striatum or CPu delineated with the green contour line in Figure 4A–C and Figure 5G) and nearby dlSVZ at PN21, and the dlSVZ and NSCN within this span at PN17/18, were investigated in the sampled sections. For all regions of interest in the PN17/18 and PN21 brains, each sampled section was viewed under a 20× objective lens on an Olympus BX51 light microscope (yielding a real viewing magnification of ×445). This microscope was fitted with an automated mechanical stage, an electronic microcator (to measure height in the z-plane), and a video camera that projected the image onto an adjacent computer monitor. Across all rats, 5–18 sections were sampled in the CPu, NSCN, and dlSVZ per rat (see Table 1 for the specific details per experiment).

Using Stereoinvestigator software installed in the computer, the Vref of the CPu, NSCN and dlSVZ was measured using the Cavalieri estimator function. Specifically, the boundaries of each region of interest were outlined in each sampled section by applying the definitions defined above. A grid of systematic points with a specific interpoint distance (e.g., of 200 µm, see Table 1) was then superimposed, randomly, over the outlined region in each section. The software counted each point of the grid that fell on/within each region of interest in each sampled section and summed the points (∑P) for each entire hemisphere. The Vref was then determined [6,49,50] from ∑P × a(p) × *t* × s, where a(p) is the real area that each point represents, *t* is the section thickness (of 5 µm), and s is the section interval (of 30 for example for the CPu, see Table 1 for the specific details for each region of interest).

#### 2.6.2. Measurement of the Nv

The Abercrombie method was used to measure the cell density (i.e., number of cells per subvolume, Nv) of immunostained DARPP-positive MSNs in the CPu at PN21. Abercrombie’s method was also used to measure the N_v_ of immunostained BrdU-positive dividing cells in the NSCN and IBA-1-positive microglia cells in the dlSVZ and CPu at PN17/18. Abercrombie’s method was used as it is less time-consuming than other methods (e.g., the physical disector method, [6]). For this method, each sampled section (from which the Vref was calculated using Cavalieri’s method) was visualized and analyzed under a 100× oil-immersion objective lens on an Olympus BX51 light microscope (yielding a real viewing magnification of ×2250). These sampled sections were also imaged for subsequent measurement of the somal or nuclear diameter (see below). Again, using the Stereoinvestigator software, the grid of systematic points obtained from the Cavalieri’s estimator function were superimposed on the region of interest, with each point used to determine each subvolume to sample. Using systematic sampling after a random start, an unbiased sampling frame [53] of 60 × 60 µm (DARPP-32) for the CPu, 20 × 20 µm (IBA-1) for the dlSVZ, and 40 × 40 µm (IBA-1) for the CPu (Table 2), was used to calculate an average uncorrected N_v_ of each specific cell type of interest. The number of nuclei that came into focus through the 5 µm height of the section, and that fell on the inclusion lines and within the unbiased sampling frame, were counted. The volume of the sampling subvolume was determined from the area (A) of the sampling frame (corrected for magnification) multiplied by the measured section thickness (*t*) (of 5 µm in this study). The average was obtained from each sampled subvolume and section. For the NSCN, all labeled BrdU-positive cells were counted in each sampled section. For the CPu every 10th or 30th area of each section was sampled, whereas for the dlSVZ every 3rd or 30th area of each section was sampled (Table 2). This ensured that at least 100–200 cells were counted to achieve reliable cell counts [54]. To determine the Nv, the total cell count for each brain was summed and then divided by the brain’s total number of sampled subvolumes.

For the PN21 experiment, the striatal MSNs were identified in the sections by their orange-DARPP-stained cytoplasm (from AEC staining) and large visible unstained nuclei (Figure 4D; see also ref. [6]). In double-labeled cells, the staining of BrdU in the nucleus was brown (from DAB staining, Figure 4D). At PN17/18, immunostained cells in the NCSN had either a black nucleus (from DAB staining, Figure 5C) or an orange nucleus (from AEC staining in initial experiments). These cells were counted as BrdU-positive cells. In a second pair of immunostained systemically sampled sections through the brain of each PN17/8 rat, cells in the dlSVZ and CPu that had a dark brown/black circumferential, or almost circumferential, soma (from DAB immunostaining) and had a pale nucleus with a minimum diameter of 3 µm, were counted as IBA-1-positive microglial cells (Figure 5E,F,H,I). These cells also usually had at least one brown/black immunostained process connected to each identified immunostained soma. For all experiments, the cell nucleus was used as the counting unit [6].

The different cell types counted were likely to have a diameter that was larger than the 5 µm depth of each section. Therefore, it is probable that the uncorrected N_v_ was an overestimate of the N_v_ for each measured subvolume. This was corrected for by using Abercrombie’s method, which considers the size of the cells [55]. For this method, digital images taken of all sampled subvolumes were used to measure the somal or nuclear (for the NSCN) diameter of a subset of counted cells. Using Fiji software [56], the maximum diameter in the long-axis was measured as well as the minimum diameter perpendicular to this. The mean of the maximum and the minimum diameter was then calculated for each cell. The average measured mean diameter (D in µm) for all sampled cells was then calculated for each animal. The corrected N_v_ for each rat was then determined from N_v_ = (uncorrected N_v_) × [t/(t + D)], where uncorrected N_v_ = ∑N/∑F from all sampled subvolumes, ∑N is the sum of the cell type counted, and ∑F is the sum of all frames (i.e., subvolumes) examined [6]. While the N_v_ provides a measure of cell density, it may not accurately represent the total (i.e., absolute) number of cells across the region of interest (i.e., the entire CPu, NSCN, and dlSVZ, [6,55,57]).

#### 2.6.3. Measurement of the Absolute Number (*N*)

Hence, the *N* of each cell type of interest within each region of interest was also calculated by multiplying the Vref by N_v_ [6,48,49,50]. The corrected N_v_ was used in this calculation. The mean *N* for each cell type of interest for all groups were then graphed and statistically analyzed.

### 2.7. Statistical Analyses

The coefficient of error (CE) for the Vref, N_v_ and *N* was calculated, or derived (see Results), as previously described [6]. Body weight was analyzed using repeated measures one-way ANOVA (IBM SPSS Statistics, version 25). Statistical analyses of the brain weights and the stereological data were carried out in GraphPad Prism using either a one-way ANOVA (for the four groups being compared in the MSN experiment) or a paired two-tailed Student’s *t*-test (for the two groups being compared in the NSCN and IBA-1 experiments). Prior to completing these analyses, it was checked and confirmed that the data were normally distributed. If a one-way ANOVA for a specific measurement yielded a statistical difference (i.e., *p* < 0.05), then a Holm–Šídák’s post-hoc test, corrected for multiple comparisons, was carried out to identify the groups that yielded the statistical difference.

## 3. Results

At 24 h after a delayed administration of AD-MSCs×2 (Figure 3A(i,ii)), a substantial number of BrdU-immunostained AD-MSCs was discovered in the brain (Figure 6). The results for these BrdU-labeled AD-MSCs were similar in the five PN17/18 rat pups from the two separate litters. It was hypothesized that the AD-MSCs would be located in the blood vessels and mainly in the dlSVZ [58,59]. However, they were generally not found in the blood vessels. They were predominantly located within the tissue of the dlSVZ (Figure 6A,B), where they presumably had a major influence. The AD-MSCs were also present in the medial-SVZ (Figure 6A,B) which is considered the NSCN (Figure 5C), where the intrinsic stem cells are proliferating [60]. The AD-MSCs were also spread throughout the entire subependymal layer of the LV known as the SVZ (Figure 6D; black arrow in Figure 6E). Furthermore, labeled AD-MSCs were present in the ependymal layer of the LV beyond the dlSVZ (white arrows; Figure 6E). Interestingly, there were also labeled AD-MSCs evident in other brain regions (i.e., the CPu and lateral septum, Figure 6A,B,D,E). This suggests that the AD-MSCs passed through the lining of the blood vessels and migrated into these brain regions (see also the Discussion). The observed location of AD-MSCs within the brain suggests they can influence neurological repair processes after a hypoxic-ischemic insult.

**Figure 7:**
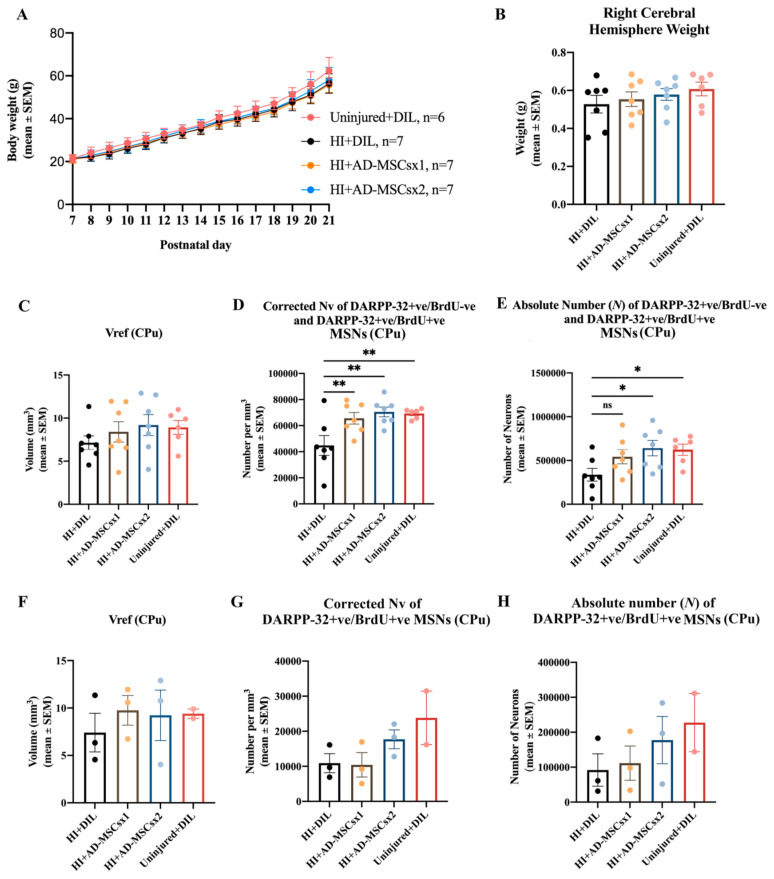
Results from the experiment on medium-spiny neurons (MSNs) in the caudate-putamen (CPu). Pups were exposed to one of the following conditions: hypoxia-ischemia (HI) and diluent (HI + DIL), single treatment with adipose-derived-mesenchymal stem cells (HI + AD-MSCs×1), double treatment with AD-MSCs (HI + AD-MSCs×2) or no HI and diluent (i.e., an uninjured control condition). (**A**,**B**) Effect of delayed treatment with either AD-MSCs, or diluent, on postnatal day (PN) 14 and PN16, after perinatal hypoxia-ischemia (HI) at PN7, on (**A**) the average body weight from PN7 to PN21, (**B**) the average right cerebral hemisphere brain weight at PN21, and (**C**–**E**) the stereological parameters relevant to the measurement of all MSNs within the CPu, including (**C**) the total reference volume (Vref) of the CPu, (**D**) the corrected density (Nv) of dopamine and cyclic AMP-regulated phosphoprotein (DARPP-32)-positive/bromodeoxyuridine (BrdU)-negative and DARPP-32-positive/BrdU-positive MSNs in the CPu, and (**E**) the absolute number (*N*) of DARPP-32-positive/BrdU-negative and DARPP-32-positive/BrdU-positive for each group. All data in C-E are from the right cerebral hemisphere at PN21. (**A**–**E**) *n* = 6/7 per group. In a subset of these animals (**F**–**H**, *n* = 2/3 per group), the effect on progenitor DARPP-32-positive/BrdU-positive MSNs was also investigated. The stereological results relevant to this measurement of MSNs are indicated for (**F**) the total reference volume (Vref) for the CPu, (**G**) corrected density (Nv) of DARPP-32-positive/BrdU-positive MSNs in the CPu and (**H**) the absolute number (N) of DARPP-32-positive/BrdU-positive MSNs for each group. Note that three BrdU injections were administered daily, starting at 7 days after the induction of HI, to permit tracking of the differentiation of neuroblasts. The body weight data were statistically analyzed using used repeated measures ANOVA. The brain weight and stereological data were statistically analyzed used one-way ANOVA and Holm–Šídák’s post-hoc test corrected for multiple comparisons. * *p* < 0.05, ** *p* < 0.01. ns, not statistically significant. See also the text in the Results for more details on the statistical outcomes.

**Figure 8:**
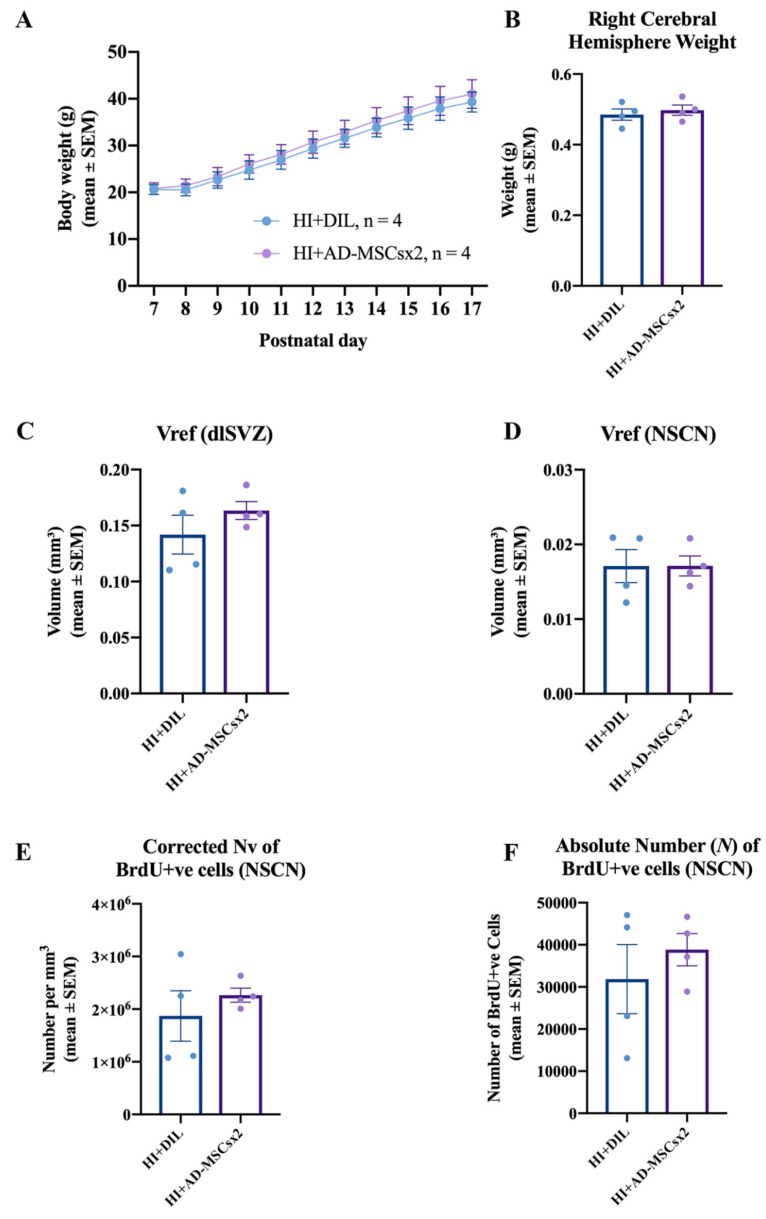
Results from the experiment on cellular proliferation. Pups were exposed to either hypoxia-ischemia (HI) and diluent (HI + DIL) or HI and double treatment with adipose-derived-mesenchymal stem cells (HI + AD-MSCs×2). (**A**–**F**) Effect of delayed double treatment with either AD-MSCs or diluent on postnatal day (PN) 14/15 and PN16/17, after perinatal HI at PN7/8, on (**A**) the average body weight from PN7/8 to PN17, (**B**) the average right cerebral hemisphere brain weight at PN17/18, (**C**) the average volumetric data of the dorsolateral subventricular zone (dlSVZ), (**D**) the average volumetric data of the neural stem cell niche (NSCN) region within the dlSVZ, (**E**) the average neuronal density (Nv) of bromodeoyuridine (BrdU)-positive cells within the NSCN, and (**F**) the average absolute neuronal number (*N*) of BrdU-positive cells within the NSCN (HI + DIL: *n* = 4; HI + AD-MSCs×2: *n* = 4). All data are from the right cerebral hemisphere at PN17/18. The body weight data were statistically analyzed using used repeated measures ANOVA. The brain weight and stereological data were statistically analyzed using a paired two-tailed Student’s t-test. See also the text in the Results for more details on the statistical outcomes.

Body weight from PN7/8 to PN17, or to PN21, was measured daily to monitor the well-being of the animals. There was a significant increase in average body weight (repeated measures ANOVA; location experiment: F (1,10) = 813.817, *p* < 0.0001; MSN experiment: F (1,14) = 1617.811, *p* < 0.0001, Figure 7A; neurogenesis and microglial experiment: F (1,10) = 378.331, *p* < 0.0001, Figure 8A) for all groups. It is noted that there was no overall increase from PN7 to PN8 (e.g., Figure 8A) for the HI animals. This is likely to be due to the surgery and then the exposure to hypoxia on PN7. No significant differences in the average body weight were present between the groups over time (repeated measures ANOVA; MSN experiment: F (1,3) = 1.579, *p* < 0.222, Figure 7A; neurogenesis and microglial experiment: F (1,1) = 0.267, *p* < 0.624, Figure 8A). There was no morbidity or mortality following the injections with diluent, AD-MSCs, or BrdU. Thus, all procedures yielded a survival rate of 100%. These results indicate the relative safety of treatment with AD-MSCs.

Right cerebral weights were compared between experimental groups, to provide an indication of their pathological, ischemic state [44,45]. For the MSN experiment, at PN21, there was no significant difference in the average weight of the right cerebrum between the HI-diluent, HI-MSCsx1, HI-MSCsx2 and uninjured control groups (F (3,23) = 0.7606 *p* = 0.5277, one-way ANOVA, Figure 7B). For the neurogenesis and microglial experiment, at PN17/18, there was no significant difference in the average weight of the right cerebrum between the HI-diluent and HI-MSCsx2 groups (paired two-tailed Student’s *t*-test, *p* = 0.6314, Figure 8B). In contrast, statistical differences were observed in the direct stereological measurement of the absolute number of striatal MSNs and microglia (see below). Hence, the right cerebral weight did not reliably reflect the absolute number of cells, nor the pathological state, in these experiments on moderate neonatal hypoxia-ischemia.

The stereological data for the MSN experiment are summarized in Figure 7. When all four groups from all five litters (i.e., pups from each litter which received, or did not receive, BrdU injections) were compared, there was no significant difference in the Vref of the striatum (one-way ANOVA, F (3,23) = 0.7786, *p* = 0.5180; Figure 7C). There was a significant difference in the N_v_ of all striatal DARPP-32 neurons (i.e., both DARPP-32-positive/BrdU-positive and DARPP-32-positive/BrdU-negative striatal MSNs) across the four groups (one-way ANOVA, F (3,23) = 5.798 *p* = 0.0042; Figure 7D). Post-hoc comparisons revealed that the N_v_ of the HI + MSCs×1 group was significantly higher compared to the HI + Dil group (Holm–Šídák’s multiple comparisons test, *t* (3,23) = 3.000 *p* = 0.0190). There was also a significant increase in the corrected N_v_ of MSNs in the CPu of AD-MSCs×2-treated animals compared to their diluent-treated littermates (Holm–Šídák’s multiple comparisons test, *t* (3,23) = 3.705 *p* = 0.0035). As expected, there was also a significant increase in the corrected N_v_ of MSNs in the CPu of uninjured control animals compared to their diluent-treated littermates (Holm–Šídák’s multiple comparisons test, *t* (3,23) = 3.389 *p* = 0.0076). In summary, the N_v_ of MSNs in the CPu was significantly increased in the animals treated with AD-MSCs. This increase was to a comparable level to control uninjured animals.

For the *N* of *all* DARPP-32-positive neurons in the striatum, there was a significant difference when the four groups were compared (one-way ANOVA, F (3,23) = 3.251 *p* = 0.0402, Figure 7E). Post-hoc comparisons revealed a significant increase in the Abercrombie-corrected *N* of MSNs in the CPu of AD-MSCs×2-treated animals compared to diluent-treated littermates (Holm–Šídák’s multiple comparisons test, *t* (3,23) = 2.811 *p* = 0.0294). There was also a significant increase in the Abercrombie-corrected *N* of MSNs in the CPu of uninjured control pups compared to their diluent-treated littermates (Holm–Šídák’s multiple comparisons test, *t* (3,23) = 2.536 *p* = 0.0366). There was a 62% increase in the *N* of MSNs within the CPu of AD-MSCs×1-treated pups compared to their diluent-treated littermates, but this was not statistically significant (Holm–Šídák’s multiple comparisons test, *t* (3,23) = 1.899 *p* = 0.0701). Thus, *delayed administration with a double high-dose of adult-sourced AD-MSCs significantly increased the absolute number of DARPP-32-positive MSNs in the dorsal striatum (i.e., CPu) when compared to diluent-treated animals.*

Comparisons between the four groups in the experiment measuring the DARPP-32-positive/*BrdU-positive* striatal MSNs revealed no significant difference in the Vref (one-way ANOVA, F (3,7) = 0.2749 *p* = 0.8419, Figure 7F), corrected N_v_ (one-way ANOVA, F (3,7) = 2.447 *p* = 0.1486, Figure 7G) and the *N* of striatal MSNs (one-way ANOVA, F (3,7) = 0.9727 *p* = 0.4577; Figure 7H). Hence, double treatment with AD-MSCs did not significantly increase the number of BrdU-labeled progenitor MSNs in the CPu (see also the Discussion).

The stereological data for the NSCN cellular proliferative experiment are summarized in Figure 8. Measurement of the dlSVZ volume provided an indirect estimate of the underlying proliferative response characterizing each group [61]. There was no significant difference between the HI AD-MSCs×2-treated group and the HI diluent-treated groups dlSVZ Vref (paired two-tailed Student’s *t*-test, *p* = 0.3748; Figure 8C). Volumetric measurements of the NSCN region of the dlSVZ provided an estimate of the size of the NSCN between the different groups. Mean total NSCN volume comparisons revealed no significant difference between the diluent- and AD-MSCs×2- treated HI groups (paired two-tailed Student’s *t*-test, *p* = 0.9947; Figure 8D). The Abercrombie-corrected neuronal density (Nv) of BrdU-immunolabeled cells in the NSCN region served as an indirect measure of the true number of proliferating neural stem cells between the different groups. Comparison of the HI-AD-MSCs×2-treated group with the HI diluent-treated group showed no statistical significance (paired two-tailed Student’s *t*-test, *p* = 0.4480; Figure 8E). The absolute number of BrdU-positive cells in the NSCN region within the dlSVZ presented the most reliable measure of the true number of proliferating neural stem cells between the different groups. Comparisons between the two HI groups revealed no statistical difference (paired two-tailed Student’s *t*-test, *p* = 0.5056; Figure 8F). Thus, *enhanced proliferation of cells within the NSCN of the dlSVZ is not a primary neurorepair mechanism in this study.*

**Figure 9:**
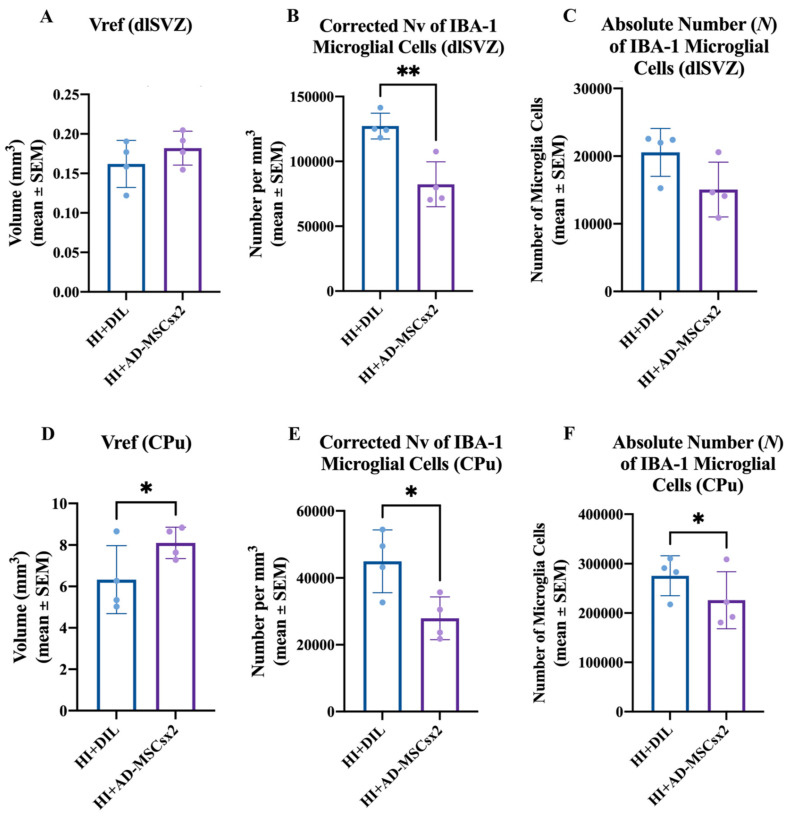
Results from the experiment on microglia. Pups were exposed to either hypoxia-ischemia (HI) and diluent (HI + DIL) or HI and double treatment with adipose-derived-mesenchymal stem cells (HI + AD-MSCs×2). (**A**–**F**) Effect of delayed double treatment with either AD-MSCs or diluent on postnatal day (PN) 14/15 and PN16/17, after perinatal HI at PN7/8, on microglia in the dorsolateral subventricular zone (dlSVZ, **A**–**C**) and in the caudate-putamen (CPu, **D**–**F**). For the dlSVZ, the relevant stereological data are (**A**) the total reference volume (Vref), (**B**) the corrected numerical density (Nv) of microglial cells, and (**C**) the absolute number (*N*) of microglial cells. For the CPu, the relevant stereological data are (**D**) the total reference volume (Vref), (**E**) the corrected numerical density (Nv) of microglial cells, and (**F**) the absolute number (*N*) of microglial cells (HI + DIL: *n* = 4; HI + AD-MSCs×2: *n* = 4). All data are from the right cerebral hemisphere at PN17/18. The brain weight and stereological data were statistically analyzed using a paired two-tailed Student’s t-test. * *p* < 0.05, ** *p* < 0.01. See also the text in the Results for more details on the statistical outcomes.

The stereological data for the microglial experiment are summarized in Figure 9. There was no significant difference in the Vref of the dlSVZ when the AD-MSCs×2- and diluent-treated HI pups were compared (paired two-tailed Student’s *t*-test, *p* = 0.4867, Figure 9A). For the total CPu volume, there was a statistically significant increase in the AD-MSCs×2-treated pups compared with the diluent-treated HI pups (paired two-tailed Student’s *t*-test, *p* = 0.049, Figure 9D). The Abercrombie method revealed a significant *decrease* in the corrected N_v_ of IBA-1 positive cells in AD-MSCs×2-treated animals compared to diluent-treated animals in both the dlSVZ (paired two-tailed Student’s *t*-test, *p* = 0.0017; Figure 9B) and the CPu (paired two-tailed Student’s *t*-test, *p* = 0.010, Figure 9E). Comparisons between the HI AD-MSCs×2 group and the HI diluent group also revealed a 18.0% *decrease* in the *N* of *CPu* IBA-1 cells in the HI AD-MSCs×2 group compared to the diluent-treated group. This was statistically significant (paired one-tailed Student’s *t*-test, *p* = 0.042; Figure 9F). A one-tailed *t*-test was used because it was hypothesized, based on the literature [11,23,62,63], that stem cell treatment would decrease the proliferative inflammatory response by microglia. When the *N* of IBA-1 immunolabeled microglial cells in the *dlSVZ* was compared between the AD-MSCs×2-treated and diluent-treated HI pups, a 27% decrease was noted in the stem cell-treated group that was not statistically significant (paired two-tailed Student’s *t*-test, *p* = 0.1258, or paired one-tailed Student’s *t*-test, *p* = 0.0629; Figure 9C). Hence, *delayed administration with a double dose of AD-MSCs significantly decreased inflammation in the CPu.*

The measured mean somal or nuclear diameter of MSNs in the CPu, proliferating cells in the NSCN, and microglia in the dlSVZ and CPu is indicated in Table 3. There was only one significant difference between the groups. The average somal size of microglia in the CPu was smaller in the HI-MSCsx2 group compared with the HI-Diluent group (Table 3). The measured diameters are within the expected range for each cell type [6,36,64]. The finding of decreased somal size may reflect a change in the sub-type of microglia (e.g., more M2-microglia) within the CPu in response to treatment with AD-MSCs. This requires investigation in a future study.

The mean CEs for the Vref measurements for the four groups ranged from 1.2% to 1.7%. A mean CE of less than 10% or less is generally considered to yield a reliable estimate since the variation (or CV = SD/mean) between animals for the parameter of interest is usually at least 10–15% [65]. For the N_v_ and *N* of neurons measured using Abercrombie’s and an unfolding method, several hundred nuclear profiles (ranging from 90 to 200) were generally measured for the somal or nuclear diameter per cell type per animal. This generally fulfilled guidelines and statistical outcomes were not jeopardized [6,66]. Thus, the stereological measurements were reliable.

## 4. Discussion

This study showed that, after neonatal rat hypoxia-ischemia, delayed administration with a double high-dose of adult-sourced AD-MSCs significantly increased the absolute number of DARPP-32-positive MSNs in the dorsal striatum (i.e., CPu) when compared to diluent-treated animals. This treatment strategy with AD-MSCs also significantly increased the absolute number of striatal MSNs to normal uninjured levels. The rat hypoxia-ischemia occurred at PN7 and the delayed double treatment with AD-MSCs was administered 7 and 9 days later (i.e., on PN14 and on PN16). There was one treatment per day and 1,070,000 AD-MSCs, on average, per injection. The short-term effect on DARPP-positive MSNs was measured at PN21. To our knowledge, this is the first time that these effects have been reported after neonatal rat hypoxia-ischemia.

For the first time, potential mechanisms of action of AD-MSCs in this context were also investigated. A very similar double treatment strategy was used. Specifically, the rat hypoxia-ischemia occurred at PN7/8 and the delayed double treatment with AD-MSCs was administered 7 and 9 days later (i.e., on PN14/15 and on PN16/17). After this double treatment with AD-MSCs, there was a significant decrease in the absolute number of IBA-1-positive microglia within the CPu, but not the dlSVZ (*p* = 0.063), at PN17/18. Hence, at this time point, the AD-MSCs were more effective in decreasing the inflammatory response in the CPu relative to the dlSVZ. The same double treatment strategy yielded no difference in the absolute number of proliferating progenitor cells in the NSCN of the dlSVZ at PN17/18. Thus, while increased neurogenesis or cellular proliferation has been proposed as a neurorepair mechanism triggered by MSCs [6,23], the evidence from this study suggests that the primary repair mechanism facilitated by AD-MSCs within 3 days of their first administration, and at 10 days after neonatal rat hypoxia-ischemia, is one of significantly decreased inflammation in the CPu.

The effect of this double treatment strategy with AD-MSCs on the differentiation of progenitors into MSNs in the CPu was also investigated at PN21. It was found there was a suggestion of an increased number of differentiated progenitors, but this was not significantly different. This is discussed below in ‘Biological Considerations’.

Single treatment with AD-MSCs (i.e., AD-MSCs×1), administered at 7 days after PN7 rat hypoxia-ischemia, did not significantly increase the absolute number of MSNs in the CPu when compared to diluent-treated animals at PN21. By contrast, single treatment with BMSCs, at 7 days after PN7 rat hypoxia-ischemia, significantly increased the absolute number of MSNs in the CPu [6]. These treatments were not compared in the same experiment due to logistical constraints. Based on the current data, single delayed treatment with BMSCs appears to be more effective than AD-MSCs in increasing the absolute number of striatal MSNs after neonatal hypoxia-ischemia.

### 4.1. Methodological Considerations

Adult-sourced MSCs were used since they may have the earliest impact clinically [8]. The relative ease in accessing and isolating AD-MSCs, and their safety and lack of ethical concern [8], have made adult-sourced MSCs a feasible clinical option [67].

FBS was prescreened to ensure that it was effective in supporting the proliferation of cultured MSCs. This strategy yielded a significant increase in the absolute number of MSNs in vivo after delayed treatment with AD-MSCs (this study) and with BMSCs [6]. These results support the recommendation [68] that FBS be prescreened to optimize the probability of a positive effect of in vivo treatment with FBS-cultured MSCs.

Evidence that the cultured cells were stem cells was provided by their adherence to plastic in vitro, their expression of stem cell cluster of differentiation (CD) markers, and their differentiation into adipocytes and osteocytes in vitro. All these features are classic characteristics of stem cells [19,20,21].

For the measurement of striatal injury, the weight of the cerebral hemisphere and striatal volume are indirect measures and may not reliably reflect the absolute number of neurons present in the striatum [9,57]. Hence, the use of such measures can lead to misinterpretation of results and may not be effective in investigating putative treatments for neonatal hypoxic-ischemic brain injury [57]. For example, in this study the weight of the right injured cerebral hemisphere, or the striatal volume, did not show any significant differences when the HI-DIL, HI-AD-MSCsx1, HI-AD-MSCs×2, and the uninjured groups were compared (Figure 7B,C,F). The efficacy of HI-AD-MSCs×2 treatment was only apparent when the absolute number, or Nv, of striatal medium-spiny neurons was compared between the groups (Figure 7D,E). This was also apparent for the uninjured normal animals versus the HI + Dil group (Figure 7D,E). An effect of HI-AD-MSCs×1 treatment was only apparent when the Nv, but not the absolute number, of striatal medium-spiny neurons was compared with the HI + Dil group (Figure 7D,E). These data highlight the scientific value of stereological measurement of absolute neuronal number.

The subcutaneous route was shown to be a therapeutically effective route for administering MSCs, supporting previous findings [6]. Thus, direct administration of MSCs into the injury site is not required. In earlier studies on neonatal hypoxic-ischemic brain injury, MSCs were administered through the intracerebral, intracardiac, intravenous or nasal routes [11,12,13,17,32,69]. Administering MSCs subcutaneously, into the back of neck in this study, is minimally invasive [70]. After a subcutaneous or intraperitoneal injection, MSCs migrate inside the host and have the capacity to engraft to sites of tissue inflammation and injury [71]. Migration inside the host and localization within sites of injury occurs via the bloodstream, including via the lymphatic system to the heart for distribution to the whole body [71]. The intraperitoneal injection of MSCs is effective in other types of injury, including blindness and experimental allergic encephalitis [72,73].

It was necessary to confirm that the injected AD-MSCs were present in the brain and to investigate where they were specifically located [22]. A substantial number of labeled cultured AD-MSCs were present in the brain of rat pups at 24 h after their second administration. The same pups had been exposed to neonatal hypoxia-ischemia 10 days earlier. The presence of AD-MSCs within the brain suggests they can influence neurological repair processes after an hypoxic-ischemic insult. The injected stem cells were heavily located within the dlSVZ, including the NSCN. They were also present in the CPu and lateral septum, to a lesser extent. Hence, these are the brain regions that they could influence. These results contradicted past research which indicated that the survival of injected MSCs is extremely limited in the neonatal HI brain. Previous studies, however, examined the location of the injected stem cells in the brain 72 h after stem cell administration [23,28,63,74]. Thus, it may take a few days for the injected stem cells to perish. Collectively, this evidence suggests that the AD-MSCs only need to be present in the brain for a short time to be effective. It also suggests that the potential for long-term side-effects due to the proliferative activity of AD-MSCs is low.

### 4.2. Biological Considerations

Due to its proximity to the striatum, the SVZ is a significant area for the regeneration of MSNs. The endogenous neurogenic pathways of neural stem cells residing in the SVZ, and subgranular zones, are stimulated by acute neonatal hypoxic-ischemic brain injury [40]. This hypoxic-ischemic-induced neurogenesis in the SVZ commences at 2 days after injury and lasts for at least 2 months [40]. The neuroblasts migrate to areas of injury yet differentiate into calretinin interneurons in the striatum rather than MSNs [75]. In addition, these calretinin neurons do not survive because of the lack of trophic support [40].

In the current study on acute neonatal hypoxic-ischemic brain injury, double treatment with AD-MSCs increased the absolute number of MSNs in the CPu to normal uninjured levels, but it did not significantly increase the number of BrdU-labeled progenitor MSNs in the CPu, at PN21. Based on the literature, one proposed mechanism(s) of action of MSCs is facilitation of the differentiation of progenitors derived from the SVZ into striatal MSNs [6]. This differentiation may be triggered by the myriad of growth factors produced by MSCs, including AD-MSCs [17,18,28,59]. How then did the increase in the overall absolute number of MSNs occur? One possibility is that the BrdU label was diluted through enhanced/extensive cell division [76] within the non-NSCN component of the dlSVZ after treatment with AD-MSCs. This could have yielded fewer detectable BrdU-labeled MSNs in the CPu.

Related to this possibility is the evidence of a significantly decreased density of microglia in the dlSVZ at PN17/18. This may have enhanced the division and survival of neuroblasts that had originated in the NSCN of the SVZ and were migrating to the CPu. Previous work after adult stroke has shown that the anti-inflammatory drug indomethacin substantially increased the number of newborn surviving cells that originated from the SVZ, that a higher fraction of these cells adopted neuronal markers, and that microglial activation limited the survival of the newborn progenitor cells [76].

Another possible contributing factor is that the AD-MSCs given at 7 days, and then at 9 days, post-injury protected or rescued moderately injured MSNs that would otherwise have died. These specific MSNs would have been part of the population resident in the CPu at the time of neonatal hypoxia-ischemia. The AD-MSCs may have provided neurotrophic support for these MSNs (i.e., by restoring cellular energy and by enhancing angiogenesis). Taken together, the evidence from the literature and from this study indicates that reasonable mechanisms are possible by which the significant increase in the absolute number of MSNs in the CPu may have arisen after double delayed treatment with AD-MSCs.

There was a significant increase in the absolute number of MSNs in the CPu after single treatment with high-dose BMSCs [6], but not after single treatment with high-dose AD-MSCs (this study). This may be due to the 10% difference in the growth factor profile produced by each stem cell population [18]. It may also be due to the increased production of vascular endothelial cell growth factor and hepatocyte growth factor by AD-MSCs [17]. How the specific growth factor profiles, for AD-MSCs versus BMSCs, influence MSNs in the CPu after neonatal hypoxia-ischemia requires future investigation.

In the current study, a significant increase in the absolute number of MSNs in the CPu after double treatment with AD-MSCs, but not after single treatment, may be due to a more prolonged presence, and/or higher concentration, of stem cell-secreted growth factors after a double dose. Other studies have also reported enhanced effect sizes after double or multiple treatment with MSCs compared with single treatment [12,15].

Overall, in terms of MSNs in the CPu after neonatal hypoxia-ischemia, the current data indicate that BMSCs have the advantage of a single treatment being effective [6], but the disadvantage of harder extraction from source tissue. AD-MSCs have the advantage of easier extraction from source tissue, and the disadvantage of requiring two doses for effectiveness. Importantly, the findings that both BMSCs and AD-MSCs are effective, each in their specific way after neonatal rat hypoxia-ischemia, supports the findings from other types of brain injury (e.g., adult stroke, [77]).

Our results on body weight provide further evidence of the relative safety of therapy with MSCs after neonatal rat HI. Previous studies showed that there was no significant difference between diluent- or MSCs-administered groups when they were 6 weeks of age [32] and 3 months of age [6].

## 5. Conclusions

This study demonstrates the therapeutic efficacy of a subcutaneously administered double high-dose of adult-sourced AD-MSCs in increasing the absolute number of striatal MSNs after HI-induced brain injury in neonatal male rats. It has also provided evidence that a primary mechanism of repair triggered by AD-MSCs involves significantly decreased striatal inflammation. The results may lead to the development of clinically effective and less invasive stem cell therapies for neonatal HI brain injury.

## Figures and Tables

**Table 1:** Stereological parameters used to measure the Vref of each brain region of interest.

Experiment	Section Sampling Interval	Real Area of Each Point, a(p)	Number of Sections Analysed per Brain
CPu Vref, DARRP-32 MSN experiment, PN21	Every 30th or 40th	200 µm × 200 µm = 40,000 µm^2^	6–17
NSCN, PN17/18	Every 30th	10 µm × 10 µm = 100 µm^2^	11–18
dlSVZ Vref, NSCN experiment, PN17/18	Every 30th	50 µm × 50 µm = 2500 µm^2^	11–18
dlSVZ Vref, microglial experiment, PN17/18	Every 60th	20 µm × 20 µm = 400 µm^2^	5–9
CPu Vref, microglial experiment, PN17/18	Every 60th	40 µm × 40 µm = 1600 µm^2^	5–9

**Table 2:** Stereological parameters used to measure the Nv of each cell type of interest.

Experiment	Size of Unbiased Sampling Frame	Sampling Interval
CPu Vref DARRP-32 MSN experiment, PN21	60 µm × 60 µm = 3600 µm^2^	Every 10th
NSCN, PN17/18	Cells in entire NSCN measured	Not applicable
dlSVZ Vref, NSCN experiment, PN17/18	20 µm × 20 µm = 400 µm^2^	Every 3rd
dlSVZ Vref, microglial experiment, PN17/18	40 µm × 40 µm = 1600 µm^2^	Every 30th
CPu Vref, microglial experiment, PN17/18	60 µm × 60 µm = 3600 µm^2^	Every 30th

**Table 3:** Average (± SEM, or SD when indicated) of the nuclear or somal diameter for each group.

**PN21 MSN Experiments**	**BrdU-Positive/DARPP-Positive Neurons in CPu**	**BrdU-Negative/DARPP-Positive Neurons in CPu**	**All DARPP-Positive Neurons in CPu**
HI-DIL	8.69 ± 0.56	9.12 ± 0.56	8.91 ± 0.55
HI-MSC×1	9.29 ± 0.55	9.46 ± 0.55	9.38 ± 0.55
HI-MSC×2	9.24 ± 0.89	9.30 ± 0.39	9.27 ± 0.87
Uninjured	9.18 ± 0.24 (SD)	9.12 ± 0.17 (SD)	9.15 ± 0.04 (SD)
**PN17/18 Mechanism(s) Experiments**	**BrdU-Positive Cells in NSCN**	**IBA-1-Positive Cells in dlSVZ**	**IBA-1-Positive Cells in CPu**
HI-DIL	6.29 ± 0.20	5.02 ± 0.32	5.87 ± 0.37
HI-MSC×2	6.02 ± 0.26	5.04 ± 0.22	5.23 ± 0.31 *

* *p* = 0.0044, paired two-tailed Student’s *t* test.

## Data Availability

The data can be available on reasonable request to the corresponding author.
